# Transcriptional regulation of BRD7 expression by Sp1 and c-Myc

**DOI:** 10.1186/1471-2199-9-111

**Published:** 2008-12-27

**Authors:** Huaying Liu, Ming Zhou, Xiaomin Luo, Liming Zhang, Zhaoxia Niu, Cong Peng, Jian Ma, Shuping Peng, Houde Zhou, Bo Xiang, Xiayu Li, Shufang Li, Jiajin He, Xiaoling Li, Guiyuan Li

**Affiliations:** 1 Cancer Research Institute, Xiang-Ya School of Medicine, Central South University, Changsha, Hunan 410078, People's Republic of China; 2 The Second Affiliated Hospital of Xiang-Ya School of Medicine, Central South University, Changsha, Hunan 410078, People's Republic of China

## Abstract

**Background:**

Bromodomain is an evolutionally conserved domain that is found in proteins strongly implicated in signal-dependent transcriptional regulation. Genetic alterations of bromodomain genes contributed to the development of many human cancers and other disorders. BRD7 is a recently identified bromodomain gene. It plays a critical role in cellular growth, cell cycle progression, and signal-dependent gene expression. Previous studies showed that BRD7 gene exhibited much higher-level of mRNA expression in normal nasopharyngeal epithelia than in nasopharyngeal carcinoma (NPC) biopsies and cell lines. However, little is known about its transcriptional regulation. In this study, we explored the transcriptional regulation of BRD7 gene.

**Method:**

Potential binding sites of transcription factors within the promoter region of BRD7 gene were predicted with MatInspector Professional . Mutation construct methods and luciferase assays were performed to define the minimal promoter of BRD7 gene. RT-PCR and western blot assays were used to detect the endogenous expression of transcription factor Sp1, c-Myc and E2F6 in all cell lines used in this study. Electrophoretic mobility shift assays (EMSA) and Chromatin immunoprecipitation (ChIP) were used to detect the direct transcription factors that are responsible for the promoter activity of BRD7 gene. DNA vector-based siRNA technology and cell transfection methods were employed to establish clone pools that stably expresses SiRNA against c-Myc expression in nasopharyngeal carcinoma 5-8F cells. Real-time PCR was used to detect mRNA expression of BRD7 gene in 5-8F/Si-c-Myc cells.

**Results:**

We defined the minimal promoter of BRD7 gene in a 55-bp region (from -266 to -212bp), and identified that its promoter activity is inversely related to c-Myc expression. Sp1 binds to the Sp1/Myc-Max overlapping site of BRD7 minimal promoter, and slightly positively regulate its promoter activity. c-Myc binds to this Sp1/Myc-Max overlapping site as well, and negatively regulates the promoter activity and endogenous mRNA expression of BRD7 gene. Knock-down of c-Myc increases the promoter activity and mRNA level of BRD7 gene. The luciferase activity of the mutated promoter constructs showed that Sp1/Myc-Max overlapping site is a positive regulation element of BRD7 promoter.

**Conclusion:**

These studies provide for the first time the evidence that c-Myc is indeed a negative regulator of BRD7 gene. These findings will help to further understand and uncover the bio-functions of BRD7 gene involved in the pathogenesis of NPC.

## Background

Bromodomain consists of a motif of 59–63 amino acids [1]. It is a protein-protein interaction domain and has a specific binding affinity for acetylated lysines on N-terminal tails of histones [2]. Several studies demonstrated that bromodomain is characteristic of proteins that regulate signal-dependent, but not basal, transcription during active proliferation through modulating chromatin remodeling or acetylation of histones, therefore facilitating accession of transcription factors to chromatin [3,4]. Accummulating evidence showed that the genetic alterations of bromodomain genes contributed to the development of many human cancers and other disorders [5,6]. BRD7 is a bromodomain-containing gene identified from Nasopharyngeal carcinoma (NPC) cells by cDNA Representational Difference Analysis [7]. Due to the sequence similarity with other bromodomain containing protein, it was suggested that BRD7 may be as components of chromatin remodeling complexes and possess histone acetyltransferase activity [8]. Together with E1B-AP5, BRD7 functions as an inhibitor of basic transcription in several viral and cellular promoters in the nucleus [9]. STAAL et al. demonstrated that BRD7 protein (celtix-1) interacted with interferon regulatory factor 2 in the nucleus and associated with transcriptionally active chromatin in situ [10]. BP75, the most homologous gene of BRD7, directly interacts with Dvl-1, enhances TCF-dependent gene expression induced by Dvl-1, and induces the nuclear translocation and formation of vesicular structures of beta-catenin with Dvl-1 in a synergistic manner [11], indicating that BRD7 may play an important role in Wnt signaling. An alternative critical role of BRD7 gene arose from evidence that BRD7 gene exhibited much higher-level of mRNA expression in normal nasopharyngeal epithelia than in NPC biopsies and cell lines [12]. Over-expression of BRD7 gene in NPC cells was effective to inhibit cell growth and cell cycle progression from G1 to S phase [12-14]. But little is known about its transcriptional regulation. Our previous studies located the promoter of BRD7 gene in a 125 bp region (-293→-168 bp) [15]. In this report, we defined the minimal promoter of BRD7 gene in a 55-bp region, and explored the role of c-Myc in suppressive regulating of BRD7 expression in NPC cells. These findings will help to further understand and uncover the functions of BRD7 gene in the pathogenesis of NPC, and also provide a molecular model for studying the transcriptional regulation of other bromodomain family genes.

## Results

### Identification of BRD7 minimal promoter

**Figure 1:**
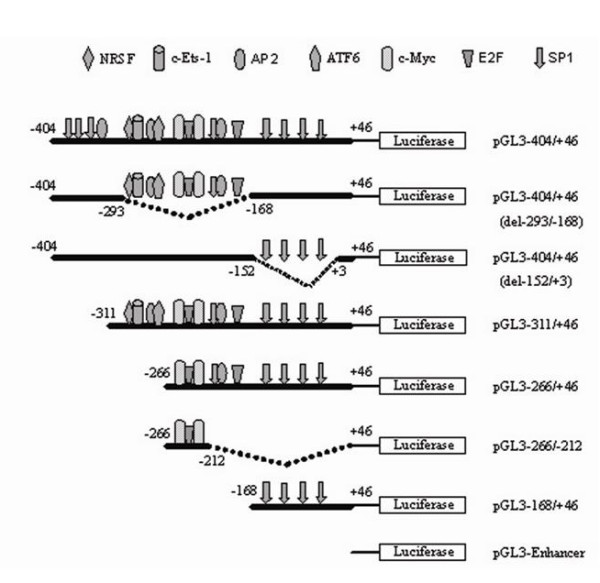
**Schematic illustration of deletion constructs of BRD7 promoter**.

**Figure 2:**
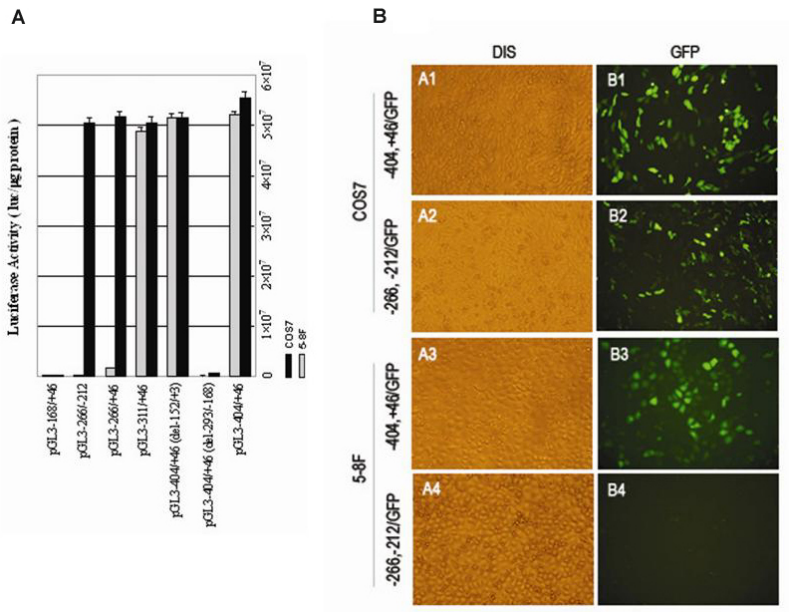
**Identificating of BRD7 minimal promoter (-266/-212)**. (A) Luciferase assay of deletion constructs of BRD7 promoter. Luciferase activity in COS7 and 5-8F cells is represented by black and gray histograms, respectively. (B) Detection of GFP fluorescence of modified construct pGL3/-266,-212/EGFP in COS7 and 5-8F cells by direct GFP fluorescence assay. Full-length modified promoter construct pGL3/-404,+46/EGFP was used as a positive control. A1, B1: COS7 cells transfected with pGL3/-404,+46/GFP; A2, B2: COS7 cells transfected with pGL3/-266,-212/GFP; A3, B3: 5-8F cells transfected with pGL3/-404,+46/GFP; A4, B4: 5-8F cells transfected with pGL3/-266,-212/GFP.

In order to define the minimal promoter of BRD7 gene, six deletion constructs were generated from full-length promoter construct pGL3-404/+46 (Fig. 1). As shown in Fig. 2A, pGL3-404/+46 expresses strong luciferase activity (approximately 5.5 × 10^7 ^luc/μg protein) in both COS7 and 5-8F cells. Two of the constructs, pGL3-311/+46 and pGL3-404/+46/(del-152/+3), show luciferase activity equally strong with the full length construct, whereas pGL3-168/+46 and pGL3-404/+46/(del-293/-168) display substantially lower luciferase activity (approximately 5.5 × 103 luc/μg protein). Surprisingly, the luciferase activities of pGL3-266/+46 and pGL3-266/-212, are as strong as that of pGL3-404/+46 in COS7 cells, but have almost no activity in 5-8F cells (Fig. 2A).

Indeed, the selective promoter activity of pGL3-266/-212 in COS7 and 5-8F cells was confirmed by direct GFP fluorescence assay. As shown in Fig. 2B, pGL3-404/+46/GFP exhibits strong GFP fluorescence both in 5-8F and COS7 cells, whereas pGL3-266/-212/GFP displays fluorescence only in COS7 cells, but shows almost no fluorescence in 5-8F cells.

### BRD7 minimal promoter activity is inversely related to c-Myc expression

**Figure 3:**
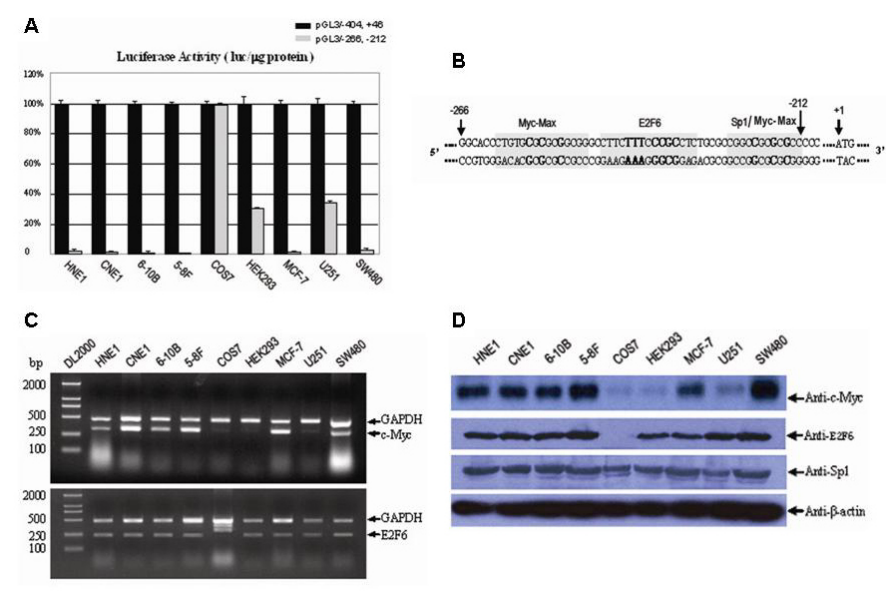
**BRD7 minimal promoter selectively functions in some cell lines with weak expression of c-Myc**. (A) Luciferase assay of pGL3/-266,-212 in different cultured cell lines. Luciferase activity of pGL3/-404,+46 and pGL3/-266,-212 is represented by black and gray histograms, respectively. (B) Bioinformatics analysis of putative cis-acting elements in BRD7 minimal promoter region -266/-212 by MatInspector program. "+1" represents the A of the initiation translation codon. (C) Detection of c-Myc, E2F6 expression in nine analyzed cell lines by RT-PCR. (D) Detection of Sp1, c-Myc and E2F6 expression in nine analyzed cell lines by western blot assay.

We next detected the activity of BRD7 minimal promoter in other cell types by using the luciferase assay. As shown in Fig. 3A, the luciferase activity of pGL3-266/-212 in COS7 cells is 99.75% of the luciferase activity of pGL3-404/+46 in COS7 cells, but only 30.56% and 33.80% of the luciferase activity of pGL3-404/+46 in HEK293 and U251 cells respectively. No luciferase activity of pGL3-266/-212 was found in HNE1, CNE1, 6-10B, 5-8F, MCF-7 and SW480 cells. Direct GFP fluorescence assay showed similar results (data not shown). These results indicate that the 55-bp promoter fragment -266/-212 of BRD7 gene selectively functions in some cell types.

To identify the upstream regulatory factor(s) of BRD7 minimal promoter, which might account for the suppressive regulation of BRD7 promoter activity in the above mentioned cell lines, we analyzed sequence -266/-212 of BRD7 gene by using MatInspector Professional program. As shown in Fig. 3B, three critical putative binding sites including the binding site for MYC-MAX (-260/-246), an E2F6 binding site (-246/-229), and a Sp1/Myc-Max overlapping site (-223/-198) were found. We, therefore, examined the endogenous expression of these transcription factors in all cell lines used in this study. As shown in Fig. 3D, Sp1 is ubiquitously expressed in all cell lines whereas E2F6 is expressed in all analyzed cells except COS7 cells. In RT-PCR assay, two different bands were amplified in COS7 cells by using the same primers to detect E2F6 expression in other eight cell lines (Fig. 3C). c-Myc was expressed strongly in the cell lines including HNE1, CNE1, 6-10B, 5-8F, MCF-7 and SW480, in which promoter -266/-212 showed almost no activity. Very weakly expressed c-Myc was detected in COS7, HEK293 and U251 cells, in which promoter -266/-212 was active (Fig. 3A, C, and 3D). These results indicate that the activity of BRD7 minimal promoter is inversely related to c-Myc expression, suggesting that c-Myc may play a critical role in regulating of BRD7 minimal promoter.

### Transcription factor Sp1 and c-Myc specifically binds to BDR7 minimal promoter

**Figure 4:**
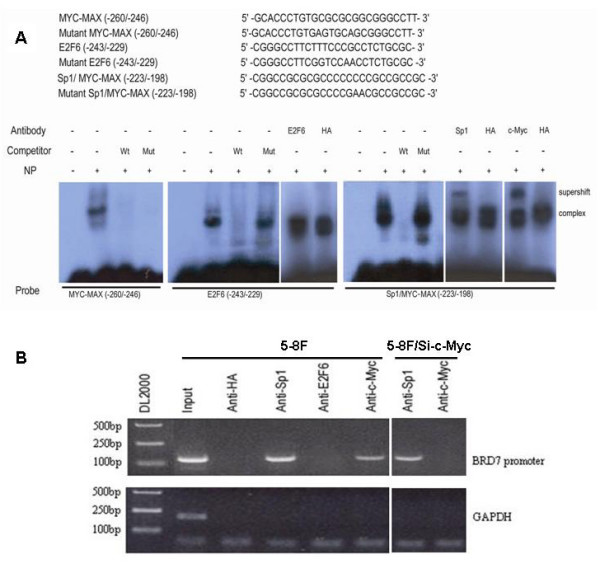
**The Sp1/Myc-Max overlapping site is specific in BRD7 minimal promoter**. (A) Top: The sense sequences of oligonucleotides used in electrophoretic mobility-shift assay. Bottom: Identification of specific E2F6 and Sp1/Myc-Max overlapping site in BDR7 minimal promoter. Oligonucleotides -260/-246, -243/-229 and -223/-198 containing a putative MYC-MAX, an E2F-6 binding site and a Sp1/Myc-Max overlapping site were respectively radiolabeled and incubated with 8 μg nuclear extracts of 5-8F cells in EMSA. For competition assay, 100-molar excess of unlabeled wild type or mutated oligonucleotide -260/-246, -243/-229 and -223/-198 were added to the reaction mixture, respectively, before the addition of radiolabeled probes. NP represents nuclear protein. In the supershift experiments, 2 μg antibody of Sp1 (Upstate Biotechnology), c-Myc (Sigma), E2F6 (Santa Cruz Biotechnology) and irrelevant anti-HA (Santa Cruz Biotechnology) was added to the reaction, respectively. (B) Transcription factor c-Myc and Sp1 are *in vivo *associated with BRD7 minimal promoter. Left panel: equivalent amounts of chromatin were immunoprecipitated in 5-8F cells by antibodies as indicated or an irrelevant antibody (Anti-HA) as a control. Right panel: equivalent amounts of chromatin were immunoprecipitated in 5-8F/Si-c-Myc cells by antibodies as indicated. PCR was performed using the indicated BRD7 primers or GAPDH primers.

To identify the direct transcription factors that are responsible for the promoter activity of BRD7 gene, EMSA and supershift assays were performed. As shown in the right panel of Fig. 4A, a strong and a weak DNA-protein complex was formed by using the radiolabeled -223/-198 fragment as a probe. The complex formation was fully suppressed by the addition of a 100-fold molar excess of cold wild type -223/-198 fragment (self inhibition), but not by 100-fold molar excess of cold mutated -223/-198 fragment as competitor. Moreover, this complex can be shifted with either anti-Sp1 or anti-c-Myc, but not with irrelevant HA antibody. These results indicate that the Sp1/Myc-Max binding site at -223 to -198 is specific. Both Sp1 and c-Myc are able to bind to the overlapping site of Myc-MAX/Sp1.

The specificity of the E2F6 binding site was analyzed with a probe coding for an E2F6 binding site spanning from -243 to -229 bp. Two DNA-protein bands found. They were totally inhibited by the addition of 100-fold molar excess of cold wild type oligonucleotide, but not by 100-fold molar excess of cold mutated oligonucleotide as competitors (Middle panel of Fig. 4A). These complexes can't be shifted with either anti-E2F6 or anti-HA, indicating that the E2F6 binding site at -243/-229 of BRD7 promoter is a functional binding site, but is not an E2F6-specific site.

Finally, we examined the specificity of MYC-MAX binding site at -260 to -246 of BRD7 minimal promoter. A strong and a weak DNA-protein complex were formed with -260/-246 as a probe, and both were completely abolished by adding either 100-fold molar excess cold wild type -260/-246 or the cold mutated -260/-246 as competitors. These data implied that the protein-DNA complexes formed at -260 to -246 were nonspecific (Left panel of Fig. 4A).

To investigate whether transcription factor Sp1, c-Myc or E2F6 interacts with BRD7 minimal promoter through their specific binding sites *in vivo*, we performed ChIP assay. Because of the high GC content (more than 80%) in the region from -266 to -212 bp, it is very difficult to design the primers for amplifying of the sequence spanning -266 to -212 bp in ChIP assay. We designed the primers for amplification the sequence spanning -274 to -162 bp. As shown in the left panel of Fig. 4B, with the DNA samples immunoprecipitated in 5-8F cells by polyclonal rabbit antibodies against Sp1 and c-Myc as templates respectively, a 133 bp DNA fragment of BRD7 promoter could be amplified by using the indicated BRD7 primers (upper bands), but none by the primers of unrelated genomic region of GAPDH gene (lower bands). In contrast, no DNA fragment of BRD7 promoter could be amplified by using DNA samples immunoprecipitated by anti-E2F6 as a template. A 133 bp DNA fragment of BRD7 promoter and a 229 bp unrelated genomic region of GAPDH gene were amplified by using the input as templates. When DNA samples immunoprecipitated with irrelevant anti-HA (negative control) were used as templates, no amplification occurred with either BRD7 primers or GAPDH primers. Moreover, we did ChiP assays using nuclear protein isolated from 5-8F/Si-c-Myc cells. As shown in the right panel of Fig. 4B, with the DNA samples immunoprecipitated in 5-8F/Si-c-Myc cells by Sp1 antibodies as templates, a 133 bp DNA fragment of BRD7 promoter could be amplified, whereas no DNA fragment of BRD7 promoter could be amplified by using DNA samples immunoprecipitated in 5-8F/Si-c-Myc cells by anti-c-Myc as templates. Taking together, we concluded that transcription factor Sp1 and c-Myc specifically bind to BRD7 minimal promoter.

### Role of the transcription factor Sp1 in the regulation of BRD7 promoter activity

**Figure 5:**
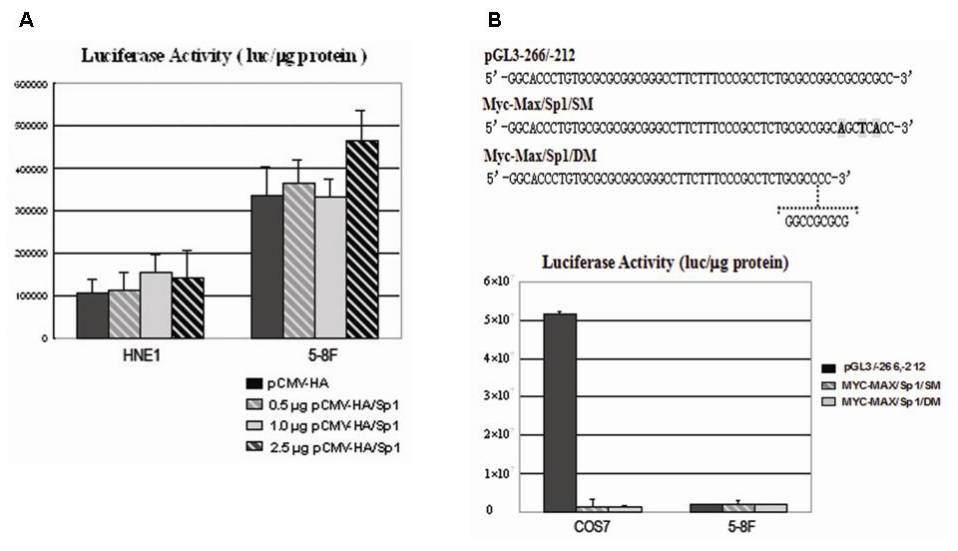
**Transcriptional factor Sp1 slightly increases BRD7 promoter activity in NPC HNE1 and 5-8F cells**. Mutation of Sp1 element decreases the activity of BRD7 minimal promoter in COS7 cells. (A) 0.5 μg pGL3/-266,-212 was cotransfected with 0.25 μg SV40 β-galactosidase vector and various indicated amounts of pCMV-HA/Sp1 into HNE1 and 5-8F cells by Lipofectamine 2000 Reagent. Luciferase activity was measured in cell extracts 38 h after transfection. The SV40 β-galactosidase vector was used for normalizing transfection efficiency. Data are the means ± S.D. of three independent experiments. (B) Top: The promoter fragment (sense) sequences to construct wild type and mutant Sp1/Myc-Max binging site reporter constructs of BRD7 minimal promoter -266/-212. Bottom: 0.5 μg promoter construct pGL3/-266,-212, Sp1/Myc-Max/SM and Sp1/DM were respectively cotransfected with 0.25 μg SV40 β-galactosidase vector into COS7 or 5-8F cells by Lipofectamine 2000 Reagent. Luciferase activity was measured in cell extracts 38 h after transfection. The SV40 β-galactosidase vector was used for normalizing transfection efficiency. Data are the means ± S.D. of three independent experiments.

To determine the role of Sp1 in regulating BRD7 minimal promoter, different amounts of expressing vector pCMV-HA/Sp1 were co-transfected with promoter construct pGL3/-266,-212 into HNE1 and 5-8F cells, respectively. As shown in Fig. 5A, overexpression of Sp1 slightly increases the promoter activity of pGL3-266/-212 in both HNE1 and 5-8F cells. Similar results were observed in CNE1, 6-10B, MCF-7 and SW480 cells (data not shown). Is this due to the invalid Sp1 binding site? To further confirm the Sp1 binding site at position -223 to -198, two mutated promoter constructs were created. Construct Sp1/Myc-Max/SM was generated by site mutation of three key nucleotides (C/A, G/T, G/A) of Sp1/Myc-Max element, whereas Sp1/Myc-Max/DM, was generated by deleting nine nucleotides of Sp1/Myc-Max element (Fig. 5B). After transfection into 5-8F cells, both Sp1/Myc-Max/SM and Sp1/Myc-Max/DM exhibited the same low activity as compared to wild type pGL3/-266,-212. In COS7 cells, wild type pGL3/-266,-212 showed strong promoter activity (approximately 5.2 × 10^7 ^luc/μg protein), but both Sp1/Myc-Max/SM and Sp1/Myc-Max/DM displayed substantially low promoter activity (Fig. 5B). All these data indicated that the Sp1/Myc-Max element spanning from -223 to -198 bp is a positive regulatory element. Mutating three key nucleotides of Sp1/Myc-Max element was sufficient to abolish Sp1 binding to this region and positively regulating of BRD7 promoter activity.

### c-Myc inhibits the promoter activity and endogenous mRNA expression of BRD7 gene

**Figure 6:**
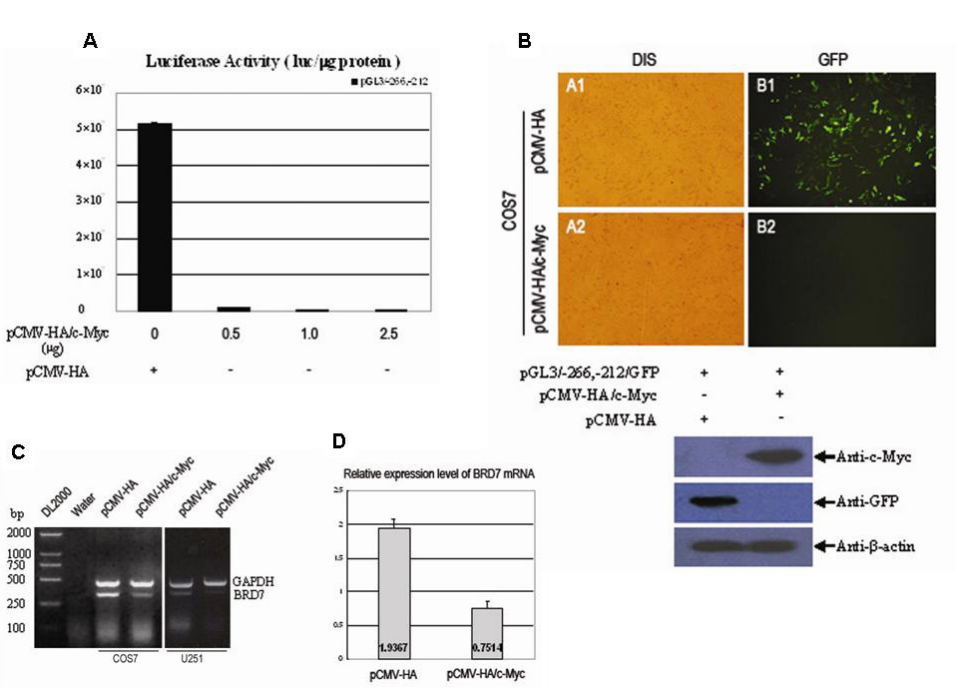
**c-Myc inhibits promoter activity and endogenous mRNA level of BRD7 gene**. (A) Luciferase assay of 0.5μg pGL3/-266,-212 in COS7 cells cotransfected with various indicated amounts of pCMV-HA/c-Myc. The SV40 β-galactosidase vector was used for normalizing transfection efficiency. Data are the means ± S.D. of three independent experiments. (B) Top: 0.5 μg modified reporter construct pGL3/-266,-212/GFP was cotransfected with 2.5 μg pCMV-HA/c-Myc or 2.5 μg pCMV-HA into COS7 cells by Lipofectamine 2000 Reagent. 38 h after transfection, the signal of EGFP fluorescence driven by promoter fragment -266/-212 was observed by using an AX-80 analytical microscope system (Olympus, Tokyo, Japan). A1, B1: COS7 cells cotransfected with pGL3/-404,+46/GFP and pCMV-HA; A2, B2: COS7 cells cotransfected with pGL3/-266,-212/GFP and pCMV-HA-c-Myc. Bottom: After examining the direct GFP fluorescence, the analyzed COS7 cells were subjected to immunoblot assay with anti-GFP as the primary antibody to detect the EGFP expression driven by promoter fragment -266/-212 with or without overexpression of pCMV-HA-c-Myc. pCMV-HA was used as control. (C) and (D) Examination of BRD7 expression in COS7 cells transfected with pCMV-HA/c-Myc by RT-PCR and Real-time PCR, respectively. pCMV-HA was used as a negative control.

To elucidate the effects of c-Myc on regulating of BRD7 minimal promoter, pGL3/-266,-212 and pGL3/-266,-212/GFP were co-transfected with various amounts of pCMV-HA/c-Myc into COS7 cells, respectively. As shown in Fig. 6A, 1.0 μg of pCMV-HA/c-Myc was sufficient to completely inhibit the promoter activity of pGL3/-266,-212. Similarly, no direct GFP fluorescence was found in cells co-transfected with pGL3/-266,-212/GFP and pCMV-HA/c-Myc (Fig. 6B), while strong direct GFP fluorescence was observed in cells co-transfected with pGL3/-266,-212/GFP and the control construct pCMV-HA (Fig. 6B). Moreover, the suppressive role of c-Myc in regulating of BRD7 mRNA expression was investigated by RT-PCR and real-time PCR. As shown in Fig. 6C, obvious down-regulating of BRD7 mRNA expression was achieved by overexpression of c-Myc in both COS7 and U251 cells as compared to overexpression of the control vector pCMV-HA. Overexpression of c-Myc decreased more than 43% of BRD7 mRNA expression in COS7 cells (Fig. 6D). All these data suggested that transcription factor c-Myc could significantly suppress the promoter activity and endogenous mRNA expression of BRD7 gene.

### Knockdown of endogenous c-Myc increases the promoter activity and mRNA expression of BRD7 gene

**Figure 7:**
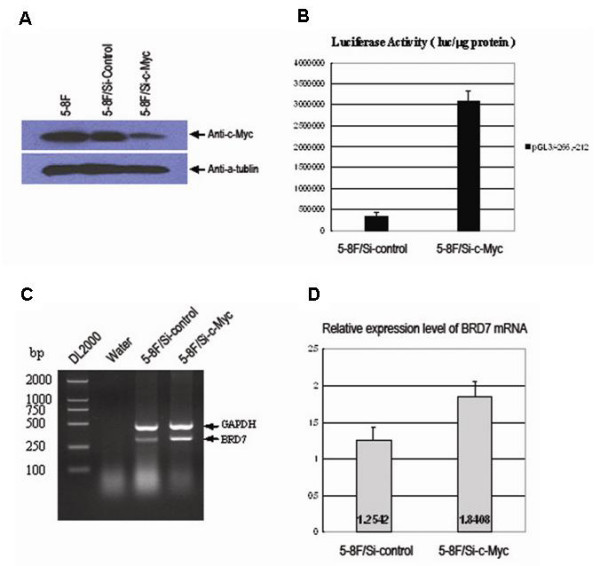
**Knockdown of endogenous c-Myc increases the promoter activity and mRNA transcription of BRD7 gene**. (A) Detecting of c-Myc expression level in 5-8F/Si-c-Myc and 5-8F/Si-control cells by immunoblot analysis. The anti-c-Myc was used as primary antibody. The expression of α-tubulin was used as an internal control. (B) Luciferase assay of pGL3/-266,-212 promoter activity in 5-8F/Si-c-Myc and 5-8F/Si-control cells. Luciferase activity was measured in cell extracts 38 h after transfection. The SV40 β-galactosidase vector was used for normalizing transfection efficiency. Data are the means ± S.D. of three independent experiments. (C) Detection of BRD7 expression in 5-8F/Si-c-Myc and 5-8F/Si-control cells by RT-PCR. The PCR products were analyzed by electrophoresis on a 2%

To further uncover the role of c-Myc in negative regulation of BRD7 expression, we employed DNA vector-based siRNA technology, by which small DNA inserts encoding short hairpin RNA against c-myc expression were cloned into pRNAT-U6.1 vector, and named as pRNA-U6.1/neo/c-Myc. pRNA-U6.1/neo empty vector was used as a negative control. pRNA-U6.1/neo/c-Myc and pRNA-U6.1/neo were transfected into 5-8F cells to establish clone pool that stably expressing siRNA against c-Myc expression. As shown in Fig. 7A, knockdown of c-Myc decreased the protein expression of c-Myc in 5-8F cells.

To identify the effects of knockdown of endogenous c-Myc expression on BRD7 promoter activity, the promoter construct pGL3/-266,-212 was transfected into either 5-8F/Si-c-Myc or 5-8F/Si-control cells. As shown in Fig. 7B, BRD7 promoter activity increases ten folds in 5-8F/Si-c-Myc cells, indicating that knock-down of endogenous c-Myc increases the promoter activity of BRD7 gene.

We, next, examined the effect of knockdown of c-Myc on endogenous BRD7 expression. mRNA expression level of BRD7 gene detected in 5-8F/Si-c-Myc cells was higher than in 5-8F/Si-control cells (Fig. 7C). The relative BRD7 mRNA level relative to GAPDH mRNA level was 1.8408 in 5-8F/Si-c-Myc cells as compared to 1.2542 in 5-8F/Si-control cells (Fig. 7D). This indicates that knockdown of c-Myc expression increased by 46% of the endogenous mRNA expression of BRD7 gene as compared to the parallel negative control.

## Discussion and conclusion

Nasopharyngeal carcinoma (NPC) is a head and neck malignancy with high occurrence in South-East Asia and Southern China [16,17]. Previous studies show that EB virus, environmental factors, and genetic susceptibility play important roles in the pathogenesis of NPC [18,19]. BRD7 is one of the genes identified from Nasopharyngeal carcinoma (NPC) cells by cDNA Representational Difference Analysis (cDNA RDA) [7]. It exhibited much higher-level of mRNA expression in normal nasopharyngeal epithelia than in nasopharyngeal carcinoma (NPC) biopsies and cell lines [12]. However, little is known about its transcriptional regulation.

In this report, we defined the minimal promoter of BRD7 gene in a 55 bp region, which is the shortest promoter identified so far in the regulatory region of BRD7 gene. Surprisingly, we found that this 55 bp region displayed high promoter activity in COS7, HEK293 and U251 cells, but exhibited extremely low activity in HNE1, CNE1, 6-10B, 5-8F, MCF-7 and SW480 cells as compared to the full-length promoter -404/+46. What causes it? MatInspector analysis revealed a MYC-MAX, an E2F6 and a Sp1/Myc-Max overlapping site in BRD7 minimal promoter, suggesting that transcription factor E2F6, c-Myc and Sp1 may be involved in regulating of BRD7 minimal promoter.

Sp1 is a well-investigated factor that regulates transcription through specific sequences in G/C-rich promoter regions and is often critical for transcriptional initiation of TATA-less promoters [20,21]. BRD7 gene contains a G/C rich promoter, in which there are several potential Sp1 binding sites and no TATA boxes. EMSA along with ChIP assays confirmed a specific Sp1/Myc-Max overlapping site spanning from -223 to -198 bp in BRD7 minimal promoter. Overexpression of Sp1 slightly increased the activity of BRD7 minimal promoter in NPC HNE1 and 5-8F cells. Is it because of the invalid Sp1 binding site? To end this point, two mutated promoter constructs including Sp1/Myc-Max/SM and Sp1/Myc-Max/DM were generated. The results showed that the Sp1/Myc-Max overlapping site in the region from -223 to -198 bp is a positive regulatory element. Mutating three key nucleotides of Sp1/Myc-Max element was sufficient to abolish its positive regulation of BRD7 promoter activity. These data confirmed the positively regulating role of Sp1/Myc-Max site in BRD7 promoter, on the other hand, inferred the possibility that the weakly positive role of Sp1 may result from the influence of some negatively regulating factors.

c-Myc is a basic helix-loop-helix leucine zipper transcription factor, and over-expressed in most human cancers [22]. c-Myc executes its multiple activities mostly through transcriptional regulation of the target genes by binding to several hundred genomic loci harboring consensus c-Myc binding sites, termed E-boxes, resulting in transcriptional activation of their adjacent genes [23]. Additionally, a large number of genes such as cell cycle/growth arrest genes gas1, p15, p21, p27, and gadd34, -45, and -153 are down-regulated by c-Myc [24-26]. In our study, c-Myc binds BRD7 minimal promoter, and negatively regulates the promoter activity and endogenous mRNA expression of BRD7 gene. Whereas knock-down of c-Myc increases the promoter activity and mRNA level of BRD7 gene in NPC 5-8F cells. From these findings, we concluded that c-Myc is indeed a negative regulator of BRD7 gene.

In conclusion, these studies showed several lines of evidences of BRD7 transcriptional mechanisms: (1) c-Myc expression is inversely corresponding to BRD7 promoter activity. (2) Sp1 binds to BRD7 promoter and slightly positively regulates BRD7 promoter activity. (3) c-Myc binds to BRD7 promoter and negatively regulates BRD7 promoter activity and mRNA expression. Together these studies provide a molecular model for c-Myc in suppressive regulation of BRD7 promoter activity and mRNA expression. These results will help to better understand the role of BRD7 gene in signal-dependent transcriptional regulation and uncover the bio-functions of BRD7 involved in the pathogenesis of NPC as well as provide new potential therapeutic target for cancer therapy from the view of overexpression of BRD7 and knock-down of c-Myc gene.

## Methods

### Cell culture and antibodies

Most of the cell lines used in this study is from the American Type Culture Collection (ATCC) cell bank. COS7 cells (African green monkey kidney fibroblast-like cell line transfected with vectors requiring expression of SV40 T antigen. ATCC CRL-1651), HEK293 cells (human embryonic kidney cell line transformed with adenovirus 5 DNA, ATCC CRL-1573), MCF-7 cells (human breast adenocarcinoma cell line, ATCC HTB-22) and U251 cells (human glioblastoma cell line, JCRB, IFO50288) were cultured in Dulbecco modified Eagle medium (DMEM) supplemented with 10% heat-inactivated fetal bovine serum (FBS), 100 U/ml penicillin and 100 μg/ml streptomycin at 37°C, 5% CO_2_. SW480 cells (human colorectal adenocarcinoma cell line, ATCC CCL-228) were cultured in RPMI1640 medium containing 10% FBS and incubated as described above. NPC CNE1, 5-8F and 6-10B are human nasopharyngeal carcinoma cells which provided by the Cancer Center of Sun Yet-Sen University, (Guangzhou, China). NPC HNE1 cells were provided by Cancer Research Institute of Central South University (Hunan, China). HNE1, CNE1, 6-10B and 5-8F cells were cultured in RPMI1640 medium containing 10% FBS.

Anti-Sp1 is from Upstate Biotechnology. Anti-c-Myc, GFP and β-actin were from Sigma. Anti-HA and anti-E2F6 are from Santa Cruz Biotechnology.

### Bioinformatics

Potential binding sites of transcription factors within the promoter region spanning from -266 to -212 bp of BRD7 gene were performed with MatInspector Professional .

### Construction of Reporter vectors

Ten reporter constructs of BRD7 promoter were created. Five of them (pGL3-404/+46/(del-293/-168), pGL3-404/+46/(del-152/+3), pGL3-311/+46, pGL3-266/+46, pGL3-168/+46) originating from the construct pGL3-404/+46 were amplified by PCR using the primers listed in Table 1. The protruding ends were digested and phosphorated in one reaction system with Blunting Kination Ligation kit (TaKaRa, Japan). The resulted blunt-ended vectors were self-ligated with T4 ligase, then transformed into *Escherichia coli *JM109. The wild type and two mutated constructs of pGL3-266/-212 were generated by synthesizing the wild type and mutated fragments of BRD7 promoter sequence (Fig. 5B) spanning from -266 to -212 bp in TaKaRa, Japan, cutting with enzyme KpnI and NheI, subcloning into pGL3-enhancer digested with the same enzymes, then naming as pGL3-266/-212, Myc-Max/Sp1/SM, Myc-Max/Sp1/DM.

**Table 1:** Primers used to construct reporter vectors originating from the construct pGL3-404/+46.

pGL3-404/+46/(del-293/-168)	Forward: 5'-GTCTTCTCGAGAGGGGCAT-3' Reverse: 5'-ACCGGAGGTGGTGCT-3
pGL3-404/+46/(del-152/+3)	Forward: 5'-GGCAAGAAGCACAAGAA-3' Reverse: 5'-CCCCTCTCGAGAAGAC-3'

pGL3-311/+46	Forward: 5'-TGCAGCACCACCTCC-3' Reverse: 5'-GTACCTATCGATAGAGAAATGTTC-3'

pGL3-266/+46	Forward: 5'-GGCACCCTGTGCGCGCGGCGGGCCTTCT-3' Reverse: 5'-CGTAGAGGTGTTTGTCCGACTTGTGC-3'

pGL3-168/+46	Forward: 5'-GTCTTCTCGAGAGGGGCAT-3' Reverse: 5'-GTACCTATCGATAGAGAAATGTTC-3'

Two other reporter constructs (pGL3-404/+46/GFP, pGL3-266/-212/GFP) were generated by replacing the luciferase gene of pGL3-enhancer with enhanced green fluorescence protein (EGFP) as follows: EGFP coding region were amplified by PCR using primers 5'-GACTTTCCAAAATGTCGTAACAACTCC-3' (forward) and 5'-GGCTCTAGATTACTTGTACAGCTCGTC-3' (reverse) with pEGFP-C_2 _as template, cut with double restriction enzyme NcoI and XbaI, then cloned into the vector fragment of pGL3/Enhancer/-404,+46 or pGL3/Enhancer/-266,-212 which were cut with the same restriction enzymes NcoI and XbaI to release the luciferase coding region.

### Construction of pCMV-HA/c-Myc, pCMV-HA/Sp1 and pRNA-U6.1/neo/Sic-Myc

The coding region of c-Myc and Sp1 were amplified by PCR using primers list in Table 2 with fetal brain cDNA library as templates, digested with enzyme EcoRI/XholI, then subcloned into pCMV-HA vectors cut with two corresponding enzymes, and named as pCMV-HA/c-Myc and pCMV-HA/Sp1.

To generate c-Myc knockdown vector, two annealed sets of oligonucleotides encoding short hairpin transcripts corresponding to nt 1357–1377 (insert 1) and nt 1716–1738 (insert 2) of c-myc mRNA (GenBank accession no. NM-002467) (21) were cloned into pRNAT-U6.1 vector (GenScript Corp.). The oligonucleotides encoding short hairpin transcripts to silence the expression of c-Myc were insert 1 (sense 5'-GATCCCGTTGGACAGTGTCAGAGTCTTCAAGAGAGACTCTGACACTGTCCAACTTTTTTCCAAA-3' and antisense 5'-AGCTTTTGGAAAAAAGTTGGACAGTGTCAGAGTCTCTCTTGAAGACTCTGACACTGTCCAACGG-3') and insert 2 (sense 5'-GATCCCGCATTTCTGTTAGAAGGAATCGTTCAAGAGACGATTCCTTCTAACAGAAATGTTTTTTCCAAA-3' and antisense 5'-AGCTTTTGGAAAAAACATTTCTGTTAGAAGGAATCGTCTCTTGAACGATTCCTTCTAACAGAAATGCGG-3'). pRNA-U6.1/Neo empty vectors were used as control. All the DNA inserts were synthesized in TaKaRa cooperation, Dalian, China, subcloned into the pRNA-U6.1/Neo vector (GenScript, USA) cut with BamH I and Hind III, then transformed into competent JM109 cells. Positive clones were sequenced to verify the correct inserts, then named as pRNA-U6.1/neo/Sic-Myc.

**Table 2:** Primers used to amplify the coding region of c-Myc and Sp1 gene.

pCMV-HA/c-Myc	Forward: 5'-ACAGAATTCACATGCCCCTCAACGTTAGCTTCAC-3' Reverse: 5'-TTTCTCGAGTCCTTACGCACAAGAGTTCCGTAGCTG-3'
pCMV-HA/Sp1	Forward: 5'-TTTGAATTCCTGCCACCATGAGCGACCAAGAT-3' Reverse: 5'-CACCTCGAGGCCTGATCTCAGAAGCCATT-3'

### Establishing of clone pools that stably expresses SiRNA against c-Myc expression

24 h prior transfection, 1 × 10^6 ^5-8F cells were seeded in 6-well plate, then transfected with 1 μg of pRNA-U6.1/neo/c-Myc and control vector pRNA-U6.1/neo by Lipofectamine 2000 Reagent (Invitrogen) according to manufacturer's instructions. 72 h after transfection, the cells were grown in 1640 medium containing 10% fetal bovine serum and 350 μg/ml G418 for 2 weeks to screen positive clone pools, and named as 5-8F/Si-c-Myc and 5-8F/Si-control.

### Luciferase assay

4 × 10^5 ^cells were seeded in each well of 12-well plates 24 h prior transfection, then transfected with 0.5 μg of various BRD7 promoter constructs and 0.25 μg SV40 β-galactosidase vector per well for normalizing transfection efficiency by Lipofectamine 2000 Reagent (Invitrogen) according to manufacturer's instructions. To detect the effects of transcriptional factor Sp1 and c-Myc on the promoter activity of BRD7 gene, cultured cells were transfected with 0.5 μg BRD7 promoter constructs, 0.25 μg SV40 β-galactosidase vector and various amount pCMV-HA/Sp1 and pCMV-HA/c-Myc, respectively. Firefly luciferase activity was measured in cell lysates 38 h after transfection by using Luciferase Assay kit (Promega). β-galactosidase activity was measured in cell lysates by β-galactosidase Enzyme Assay System (Promega). Experiments were repeated at least three times with three replicates per sample for each experiment. Results are normalized against β-galactosidase activity.

### Detection of direct GFP fluorescence

Cells were seeded in 12-well plates to 70–80 confluent on the day of transfection, then transfected with 0.5 μg of promoter reporter constructs pGL3/-404,+46/EGFP or pGL3/-266,-212/EGFP per well by Lipofectamine 2000 Reagent. To detect the effects of transcriptional factors c-Myc on the promoter activity of BRD7 gene, cultured cells were transfected with 0.5 μg BRD7 promoter constructs which expressed EGFP and various amounts of pCMV-HA/c-Myc. pCMV-HA was used as control. The signal of EGFP fluorescence was observed 38 h after transfection by an AX-80 analytical microscope system (Olympus, Tokyo, Japan).

### Immunoblot assay

Cultured Cells were harvested with a lysis buffer consisting of 50 mM Tris-HCl (pH7.5), 0.1% sodium dodecyl sulfate (SDS), 1% Triton X-100, 150 mM NaCl, 1 mM dithiothreitol, 0.5 mM EDTA, 0.1 mM phenylmethylsulfonyl fluoride, 12 mg/ml leupeptin, 20 mg/ml aprotinin, 100 mM sodium vanadate, 100 mM sodium pyrophosphate and 1 mM sodium fluoride. The concentrations of the protein extraction were determined by using Bradford assay (Bio-Rad, Hercules, CA) with bovine serum albumin as a standard. Equal amounts of total protein were loaded in each lane of an SDS-PAGE gel (12% acrylamide). After completion of gel electrophoresis, protein was transferred to nitrocellulose membrane (PALL) for 1.5 h using a blotting apparatus. The immunoblots were incubated sequentially with primary antibodies and horseradish peroxidase-coupled secondary antibodies. Signals were generated by chemiluminescence with ECL substrate reagent (Pierce).

### Electrophoretic mobility shift assay (EMSA)

Nuclear protein was prepared by using NR-PER Nuclear and Cytoplasmic Extraction Reagents (Pierce Biotechnology) according to the instructions of the manufacturer. EMSAs were performed according to the gel shift assay system (Promega) with minor modifications. Complementary oligonucleotides including oligos spanning the potential binding sites of transcription factor Myc-MAX, E2F6 and Sp1 in BRD7 promoter region were synthesized in TaKaRa Biotechnology Company. Eight micrograms of each complementary oligo was boiled for 10 min, cooled for 3 h at room temperature, and left overnight at 4°C. Probes were radiolabeled with [γ-^32^P]ATP (30–50 μCi, 3000 Ci/mmol) by 10 U T4 Polynucleotide kinase and purified on a G25 Sephadex column. Nuclear protein (8 μg in final reaction volumes of 10 μl) from 5-8F cells was incubated for 20 min at room temperature in the presence or absence of a 100-fold molar excess (relative to the radioactive substrate) of wild type or mutated oligonucleotide duplex competitors. Then 20–30,000 cpm of labeled probe oligonucleotide duplexes was added to the reaction. After another 20 min of incubation at room temperature, the samples were analyzed by electrophoresis through a 6% polyacrylamide gel run in 0.5 × Tris-borate-EDTA for 40 min at 300V, followed by detection of the radioactive species by autoradiography. In the supershift experiments, 2 μg antibody of Sp1 (Upstate Biotechnology), c-Myc (Sigma), E2F6 (Santa Cruz Biotechnology) and irrelevant anti-HA (Santa Cruz Biotechnology) was added to the reaction, respectively.

### Chromatin immunoprecipitation (ChIP)

ChIP assays were performed using a kit from Upstate Biotechnology according to the recommendations of the manufacturer. After the steps of ChIP assay, the immuno-complexes were eluted by incubation for 15 min at 25°C with 200 μl of elution buffer (1% SDS, 100 mM NaHCO3, 1 mM DTT), and reversed at 65°C for 4 h. DNA fragments were extracted with phenol/chloroform and precipitated with ethanol. A fragment of the BRD7 promoter was amplified by semi-quantitative PCR with primers as follows: 5'-AGCCGCCGGCACCCTGTG-3' (forward) and 5'-GAGAAGACGGCGCGCGAGA-3' (reverse). An unrelated genomic region of GAPDH gene used as negative control was amplified by PCR with the primers as follows: 5'-CGACCACTTTGTCAAGCTCA-3' (forward) and 5'-AGGGGTCTACATGGCAACTG-3' (reverse), yielding a 228-bp product. PCR was carried out for 35 cycles by using a step cycle of 94°C for 30 s, 54°C for 50 s, 72°C for 1 min, followed by 72°C for 10 min. The PCR products were analyzed by electrophoresis on a 2% ethidium bromide-stained agarose gel.

### RT-PCR and real-time PCR

RT-PCR was performed as previously described (14). The single-stranded cDNA was amplified by using primers as follows: BRD7 primer (forward) 5'-CAAGCTCTTTAGCCAAACAAGAA-3', (reverse) 5'-TCATTCCTGAGTGCAACAGC-3'; c-Myc primer (forward) 5'-AGCGACTCTGAGGAGGAACA-3', (Reverse) 5'-TCGCCTCTTGACATTCTCCT-3'; GAPDH primer (forward) 5'-TCTAGACGGCAGGTCAGGTCCACC-3', (Reverse) 5'-CCACCCATGGCAAATTCCATGGCA-3'. PCR was carried out for 28 cycles using a step cycle of 94°C for 40 s, 58°C for 40 s, 72°C for 1 min, followed by 72°C for 10 min. GAPDH primer was added to the reactions at the end of the fifth cycle. Quantitative real-time PCR was performed with an ABI PRISM 7700 thermal cycler (Applied Biosystem) using SYBR^® ^Premix Ex Taq™ (TaKaRa, Dalian, China.) by using the primers as in the experiment of RT-PCR. The thermal cycling conditions were were a 10 minute hold at 95°C followed by 45 cycles of 95°C for 15 seconds, 55°C for 30 seconds and 72°C 30 seconds. Calculations were performed according to the cycle threshold method. The PCR results were normalized to human glyceraldehyde-3-phosphate dehydrogenase (GAPDH) expression. Results (mean ± S.E.) were expressed as fold relative to the expression of GAPDH, which was set to 1.

## Authors' contributions

HL participated in the study design, drafted the manuscript and carried out the bioinformatics analysis, EMSA and ChIP assays. MZ performed the RT-PCR and real-time PCR experiments. Xiaomin Luo generated the reporter constructs and helped the ChIP assays. LZ performed the western blot assays and helped the EMSA assays. ZN established the clone pools that stably express SiRNA against c-Myc expression. CP helped the cell culture. JM helped the bioinformatics analysis. SP was responsible for the luciferase assay. HZ helped the bioinformatics analysis. BX was responsible for the detection of direct GFP fluorescence. Xiayu Li helped cell culture. SL helped the western blot assays. JH helped the luciferase assays. Xiaoling Li was responsible for the study coordination. GL participated in the study design and assessed the data integrity. All authors helped to draft the manuscript, and to read and approve the final version.
